# Unraveling the enigmatic role of the subthalamic nucleus

**DOI:** 10.7554/eLife.100598

**Published:** 2024-07-10

**Authors:** Qianli Yang

**Affiliations:** 1 Institute of Neuroscience, Key Laboratory of Brain Cognition and Brain-inspired Intelligence Technology, Center for Excellence in Brain Science and Intelligence Technology, Chinese Academy of Sciences Shanghai China

**Keywords:** decision making, evidence accumulation, drift diffusion model, neurons, subthalamic nucleus, Rhesus macaque

## Abstract

Subpopulations of neurons in the subthalamic nucleus have distinct activity patterns that relate to the three hypotheses of the Drift Diffusion Model.

**Related research article** Rogers K, Gold JI, Ding L. 2024. The subthalamic nucleus contributes causally to perceptual decision-making in monkeys. *eLife* **13**:RP98345. doi: 10.7554/eLife.98345.

Pondering whether “to be or not to be”, or impulsively “tilting at windmills”, decision-making comes in many forms, as so eloquently demonstrated in many great works of literature. But what causes us to sometimes come to quick conclusions while we struggle to make up our minds at other times?

Making decisions involves a complex interplay between numerous neurons located in various regions of the brain. The neural circuitry involved in this process remains, however, enigmatic. To study the cognitive processes implicated in making simple two-choice decisions, researchers often employ the Drift Diffusion Model. This mathematical framework assumes that information accumulates over time until a decision threshold is reached, and has three main parameters: the drift rate (the rate information accumulates), the decision threshold (the amount of information needed to make a decision), and the non-decision time (the time taken for processes not directly related to decision-making, such as stimulus encoding and response execution; Ratcliff, 1978; Ratcliff and McKoon, 2008).

Two of the most critical brain areas involved in decision-making are the prefrontal cortex and the hippocampus. More recently, it has been proposed that a region known as the subthalamic nucleus, which is part of the basal ganglia – and is therefore involved in motor control and integration – also has an integral role. Now, in eLife, Kathryn Rogers, Joshua Gold and Long Ding from the University of Pennsylvania report new insights into how the subthalamic nucleus contributes to decision-making (Rogers et al., 2024).

Previous computational models propose three roles for the subthalamic nucleus in decision-making and how it interacts with other parts of the brain (Figure 1): the subthalamic nucleus works with the medial prefrontal cortex to set thresholds for decision-making. These thresholds determine when enough information has been gathered to make a decision, helping control impulsivity (Hypothesis 1). Through its interaction with the external segment of the globus pallidus (a component of the basal ganglia), the subthalamic nucleus helps to calibrate how different options are evaluated, making sure the choices are assessed properly (Hypothesis 2). It helps to implement step-like, all-or-none nonlinear computations to improve the basal ganglia’s efficacy in adjusting decision bounds (Hypothesis 3; Frank, 2006; Cavanagh et al., 2011; Ratcliff and Frank, 2012; Bogacz and Gurney, 2007; Coulthard et al., 2012; Green et al., 2013; Lo and Wang, 2006; Wei et al., 2015).

**Figure 1.**
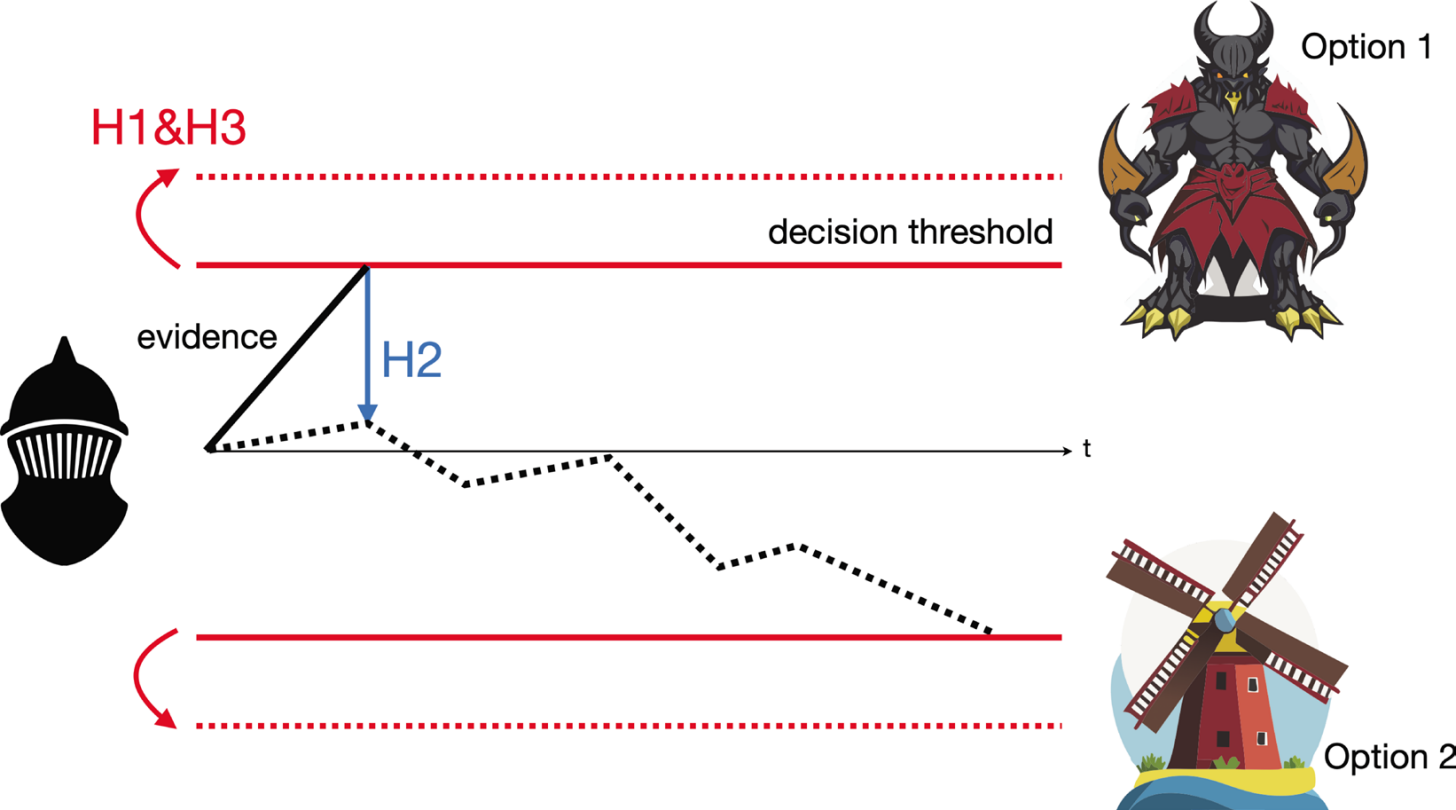
The Drift Diffusion Model illustrated by Don Quixote’s decision-making. Over time (t), Don Quixote (black mask) accumulates information that allows him to decide whether he is looking at a giant (option 1) or a windmill (option 2), and act accordingly. The subthalamic nucleus is thought to influence this decision-making process in three different ways, per the Drift Diffusion Model. First (H1), by adjusting the amount of information needed to make a decision, known as the decision threshold (represented as the gap between the two red unbroken lines increasing to the gap between the two dashed lines). Second (H2), by calibrating different pieces of evidence so they can be used to evaluate the two possibilities (whether an object is a windmill or a giant). This enables the evidence accumulation process to shift from weighting unreliable perceptual evidence too heavily (represented by the solid black line) to favoring more reliable evidence later in the decision process (represented by the dashed black line). Third (H3), by helping neural pathways sent from the basal ganglia to precisely adjust the boundaries of the decision threshold.

Image credit: Qianli Yang (CC BY 4.0).

Don Quixote’s mistaking of windmills for giants can be explained by all three hypotheses. His decision threshold may have been too low, causing him to react based on initial false evidence that the windmills’ rotating blades are the giants' flailing arms (Hypothesis 1). Similarly, he may have failed to adjust his decision threshold according to his peaceful surroundings (and thus failing to realize that he was surrounded by windmills, rather than giants (Hypothesis 3)), or to properly weigh his unreliable perception of the environment around him, which favored seeing an angry giant over a harmless windmill (Hypothesis 2).

To determine which hypothesis best explains the role of the subthalamic nucleus in decision-making, Rogers et al. recorded the activity of single neurons in the subthalamic nucleus of monkeys during a visual-saccadic decision task. In the experiment, the monkeys undertook a direction-discrimination task, during which they reported the perceived motion direction of a random dot stimulus by making a rapid eye movement (saccade) towards the corresponding choice target.

This revealed three distinct neuronal subpopulations in the subthalamic nucleus, each corresponding to one of the hypothetical models: The first subpopulation showed choice- and coherence-dependent activity that ramped up during motion viewing, consistent with the theory that these neurons pool and normalize evidence-related signals (Hypothesis 2). The second subpopulation exhibited an early, sharp rise in activity that was independent of choice and coherence during motion viewing, and gradually decreased toward saccade onset. This matches the prediction that the neurons provide an early signal to suppress immature choices (Hypothesis 1). The third subpopulation showed choice- and coherence-dependent ramping activity during motion viewing and a short burst of activity for one choice just before saccade onset. This aligns with the prediction that the subthalamic nucleus balances evidence-related signals until decision time (Hypothesis 3). This heterogeneity suggests that the subthalamic nucleus influences perceptual decision-making (where sensory information is used to guide behavior) in multiple ways.

Rogers et al. then applied electrical microstimulation (weak electric currents that affect neurons near the electrode) to the subthalamic nucleus during the task to perturb its activity. This caused the biases influencing the monkeys’ choice biases to change, reduced the influence of motion strength (i.e., the speed of the moving dot) on choices, and decreased response times. Fitting the data with a Drift Diffusion Model revealed that microstimulation of the subthalamic nucleus affected the decision threshold, evidence accumulation, as well as processes not involved in decision-making.

This groundbreaking study provides new insights into the causal roles of the subthalamic nucleus in perceptual decision-making, highlighting its involvement in modulating various aspects of the decision process through distinct neural subpopulations. These findings advance our understanding of how the basal ganglia contribute to decision-making and cognitive function.

However, it remains unclear if the subthalamic nucleus interacts with other brain regions, and if potential anatomical and/or functional alterations to this region could be implicated in neurological and psychiatric disorders. Future research could explore these issues and investigate therapy avenues that target the subthalamic nucleus for conditions involving impaired decision-making, potentially offering hope for individuals like Don Quixote.
